# MEP Latencies Predict the Neuromodulatory Effect of cTBS Delivered to the Ipsilateral and Contralateral Sensorimotor Cortex

**DOI:** 10.1371/journal.pone.0133893

**Published:** 2015-08-11

**Authors:** Gan Huang, André Mouraux

**Affiliations:** Institute of Neuroscience (IoNS), Université catholique de Louvain, Brussels, Belgium; University of Ottawa, CANADA

## Abstract

**Background:**

Recently, it was shown that the highly variable after-effect of continuous theta-burst stimulation (cTBS) of the primary motor cortex (M1) can be predicted by the latency of motor-evoked potentials (MEPs) recorded before cTBS. This suggests that at least part of this inter-individual variability is driven by differences in the neuronal populations preferentially activated by transcranial magnetic stimulation (TMS).

**Methods:**

Here, we recorded MEPs, TMS-evoked brain potentials (TEPs) and somatosensory-evoked potentials (SEPs) to investigate the effects of cTBS delivered over the primary sensorimotor cortex on both the ipsilateral and contralateral M1, and the ipsilateral and contralateral primary somatosensory cortex (S1).

**Results:**

We confirm that the after-effects of cTBS can be predicted by the latency of MEPs recorded before cTBS. Over the hemisphere onto which cTBS was delivered, short-latency MEPs at baseline were associated with an increase of MEP magnitude (i.e. an excitatory effect of cTBS) whereas late-latency MEPs were associated with reduced MEPs (i.e. an inhibitory effect of cTBS). This relationship was reversed over the contralateral hemisphere, indicating opposite effects of cTBS on the responsiveness of the ipsilateral and contralateral M1. Baseline MEP latencies also predicted changes in the magnitude of the N100 wave of TEPs elicited by stimulation of the ipsilateral and contralateral hemisphere, indicating that this TEP component is specifically dependent on the state of M1. Finally, there was a reverse relationship between MEP latency and the effects of cTBS on the SEP waveforms (50–130 ms), indicating that after-effects of cTBS on S1 are opposite to those on M1.

**Conclusion:**

Taken together, our results confirm that the variable after-effects of cTBS can be explained by differences in the neuronal populations activated by TMS. Furthermore, our results show that this variability also determines remote effects of cTBS in S1 and the contralateral hemisphere, compatible with inter-hemispheric and sensorimotor interactions.

## Introduction

Repetitive transcranial magnetic stimulation (TMS) is widely used in neuroscience research to transiently modulate the function of a given cortical structure and, thereby, probe its function. In addition, repetitive TMS is increasingly proposed as a therapeutic tool to treat various neurological and psychiatric disorders such as depression, chronic pain, migraine and stroke [1]. Depending on the parameters of stimulation, it has been suggested that repetitive TMS may either increase or decrease the excitability of the targeted cortical structure. For example, studies have shown that, when delivered over the primary motor cortex (M1), theta burst stimulation (TBS: bursts of three pulses delivered at 50 Hz and repeated at 5 Hz) may decrease M1 excitability when delivered in a continuous fashion (continuous TBS; cTBS), whereas it may increase M1 excitability when delivered in an intermittent fashion (intermittent TBS; iTBS) [2].

Despite the large number of studies aiming at characterizing the effects of repetitive TMS, the mechanisms explaining its after-effects remain largely unknown. Most importantly, it is increasingly acknowledged that the after-effects of repetitive TMS are highly variable across individuals [3], thereby markedly limiting its potential usefulness as a tool for researchers and clinicians. For example, Hamada et al. [4] showed that cTBS delivered over M1 increases the amplitude of motor-evoked potentials (MEPs) in 58% of subjects, and decreases the amplitude of MEPs in 42% of subjects. The finding that TBS may exert opposing effects in different individuals has been confirmed by several other recent studies [5, 6].

Most interestingly, Hamada et al. [4] found that the after-effects of cTBS delivered over M1 can be predicted by the latency of the MEPs recorded before applying cTBS. Specifically, they showed that short latency MEPs predicted that cTBS would exert an excitatory effect on M1, whereas long latency MEPs predicted that cTBS would exert an inhibitory effect on M1. Considering that MEP latencies are, at least in part, determined by the neuronal populations being preferentially activated by TMS (e.g. motor responses of short latency related to the direct activation of the axon hillock of pyramidal neurons vs. motor responses of long latency related to the activation of interneurons [7, 8]), the relationship between MEP latency and the after-effects of cTBS suggests that the inter-individual variability of cTBS could be driven by differences in the neuronal networks being preferentially activated by TMS. Several explanations can be put forward to explain why TMS would preferentially activate different neuronal populations across individuals. For example, these differences could be related to inter-individual differences in neuroanatomy, such as differences in the geometrical configuration of M1. Alternatively, these differences could be related to time-dependent variations in the functional state of these neuronal populations, leading these populations to be more or less sensitive to TMS at the time of stimulation.

Furthermore, studies have suggested that the effects of repetitive TMS are not confined to the cortical area targeted by TMS. For example, using positron emission tomography, Fox et al. [9] observed that 1-Hz repetitive TMS applied over M1 induces a regional increase of blood flow within the stimulated M1 and, conversely, a decrease in blood within the contralateral M1. Similarly, studies have shown that repetitive TMS over M1 tends to exert opposite effects on the ipsilateral and contralateral M1. These observations have been hypothesized to result from inter-hemispheric inhibitory connections between the left and right M1 [10–14].

Finally, several studies have shown that the combination of TMS with electroencephalography (EEG) can be used to measure local and remote brain responses to direct cortical stimulation [15, 16]. It has been suggested that these TMS-evoked brain potentials (TEPs) could be used to investigate changes in cortical responsiveness, as well as to characterize the connectivity between the stimulated region and remote regions functionally connected to the stimulated region [17, 18]. However, the functional significance of TEP waveforms remains poorly understood [19], and whether TEPs truly reflect cortical activity elicited by direct cortical stimulation remains an open question. Indeed, at least part of the TEP waveform could reflect TMS-induced artifacts such as electrode polarization artifacts due to the capacitive properties of the electrode contact, electrode movement artifacts and muscle activity artifacts due to stimulation of cranial or facial muscles. Furthermore, because the TMS pulse generates a sound that is difficult to mask as well as a tactile sensation at the site of stimulation, TEPs could receive a contribution of auditory and/or somatosensory event-related potentials (ERPs).

The first objective of our study was to replicate the recent finding showing that the latency of MEPs recorded before applying cTBS predict whether cTBS delivered over the primary sensorimotor cortex will exert an inhibitory or an excitatory after-effect on the stimulated M1. The second objective of our study was to determine whether the latency of MEPs recorded before applying cTBS would also predict the after-effects of cTBS on the excitability of the contralateral M1. Considering the inhibitory inter-hemispheric connections between the left and right M1, we hypothesized that short latency MEPs would predict reduced excitability of the stimulated M1 and increased excitability of the contralateral M1. Conversely, we hypothesized that long latency MEPs would predict increased excitability of the stimulated M1 and decreased excitability of the contralateral M1. Our third objective was to gain a better understanding of the functional significance of TEPs. For this purpose, we concomitantly recorded MEPs and TEPs elicited by stimulation of the ipsilateral and contralateral M1. We hypothesized that components of the TEP waveform that are truly related to the direct activation of M1 would be specifically modulated by cTBS delivered over M1. In contrast, TMS-induced artifacts as well as auditory- and/or somatosensory-evoked responses would be unaffected by the state of M1 and, hence, unaffected by cTBS. Finally, because the primary somatosensory cortex (S1) lies adjacent to M1, and because of the reciprocal interconnections between S1 and M1 [20], the fourth objective of our study was to assess the effect of cTBS on the state of S1 by concurrently recording somatosensory-evoked potentials (SEPs). As previous studies have suggested that the direction of the current generated inside the TMS coil may influence the respective effects of cTBS on S1 and M1 [14], we tested separately the effects of cTBS using biphasic pulses delivered in the anterior-posterior vs. posterior-anterior directions.

Importantly, all participants took part in each of the different experimental sessions, which were performed on four separate days. This design allowed us to assess whether the inter-individual variability of the effects of cTBS could be explained by fixed neuroanatomical factors (which may be expected to remain constant across sessions), or by time-varying changes in the state of the stimulated cortices (which may be expected to vary across sessions).

## Materials and Methods

### Participants

Eight healthy volunteers took part in the study (3 men and 5 women), aged from 23 to 31 years (26.11 ± 8.47 years; mean ± sd). All participants were right handed and had no history of physical or neurological illness. The study was approved by the Ethics committee of the Université catholique de Louvain (B40320096559). Written informed consent was obtained from all participants.

### Experimental design

Each participant took part in four distinct experimental sessions, performed on different days. A delay of at least 7 days separated two sessions (20 ±19 days; mean ± sd).

**Fig 1:**
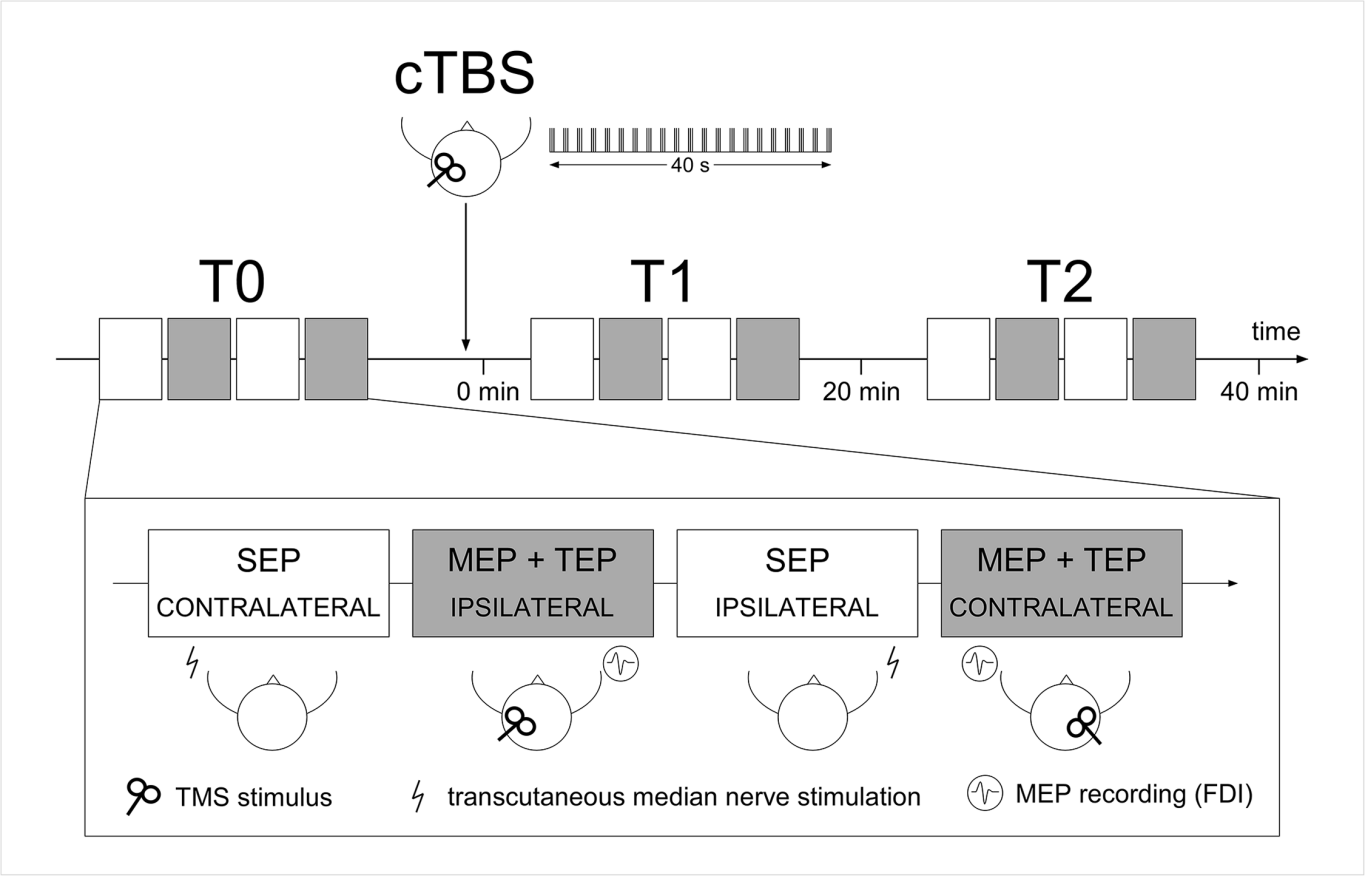
Experimental design. The experiment included three recording time points: before cTBS (T0), immediately after cTBS (T1) and 20 minutes after cTBS (T2). Each recording was completed within 20 minutes and included four blocks of stimulation. In two of the four blocks, TMS was delivered to the hand area of the left or right M1 in order to record (1) MEPs from the first dorsal interosseous muscle (FDI) of the contralateral hand and (2) TEPs. In the other two blocks, transcutaneous electrical stimuli were delivered to the left or right median nerve at the level of the wrist in order to record (3) early-latency SEPs. The order of the blocks was identical in T0, T1 and T2 for each experiment.

In each session, lasting approximately 2 hours, participants were comfortably seated on a chair with both arms placed on armrests and their head maintained in position by an adjustable headrest. The EEG was recorded continuously for the entire duration of the experiment. The participants received cTBS on either the dominant (left) or non-dominant (right) M1, using either an anterior-posterior—posterior-anterior (AP-PA) or a posterior-anterior—anterior-posterior (PA-AP) current direction (Fig 1). The order of the sessions was counter-balanced across participants.

The experiment included three recording time points: before cTBS (T0), immediately after cTBS (T1) and 20 minutes after cTBS (T2). Each recording was completed within 20 minutes and included four blocks of stimulation. In two of the four blocks, 30 single pulses of TMS (inter-stimulus interval: 5 s) were delivered to the hand area of the left or right M1 (i.e. ipsilateral or contralateral relative to the hemisphere onto which cTBS was applied) in order to record (1) MEPs from the first dorsal interosseous muscle (FDI) of the contralateral hand and (2) TEPs. In the other two blocks, 500 transcutaneous electrical stimuli (inter-stimulus interval: 0.5 s) were delivered to the left or right median nerve at the level of the wrist in order to (3) record early-latency SEPs from the ipsilateral or contralateral hemisphere relative to the hemisphere onto which cTBS was applied.

### Electroencephalogram (EEG)

The EEG was recorded using 32 scalp electrodes placed in an elastic headcap at the locations of the International 10–20 system. The electrodes have been designed specifically for the combination of EEG with TMS (Multitrodes, Brain Products GmBH, Germany). Their very flat profile allows minimizing the distance between the TMS coil and the scalp surface. Most importantly, orientation of the lead wires can be freely adjusted. This allowed positioning the wires orthogonal relative to the coil handle and then to adjust their position such as to reduce TMS-induced artifacts [21]. Finally, a thin plastic foil was wrapped around the electrode cap to reduce artifacts related to contact between the coil and the electrodes. Once impedances below 5 kΩ were ensured, the signals were amplified and digitized at 2 kHz using an Asalab HS64 EEG system (ANT Neuro, Enschede, NL). The ground electrode was positioned at AFz. A common average served as the active reference. Two electrodes placed at the upper-left and lower-right sides of the right eye were used to monitor ocular movements and eye blink artifacts.

### Transcranial magnetic stimulation (TMS)

TMS was delivered using a 75 mm figure-of-eight coil (C-B60, Magventure, Denmark) connected to a MagPro X100 MagOption magnetic stimulator (Magventure, Denmark). The stimuli consisted of a 280 μs biphasic pulse delivered either using AP-PA or PA-AP current direction. At the beginning of the experiment, the coil was positioned over the hand area of the left or right M1, at an angle 45° relative to the midline. The “hot spot” of the M1 hand area was identified by searching for the coil position at which single pulses slightly above threshold consistently produced the largest motor-evoked potential (MEP) in the FDI muscle of the contralateral hand [22]. This location was marked on the cap to provide a reference point. The position of the coil was maintained using an articulated mechanical arm, and the position of the head was maintained using the adjustable headrest of the dentist chair. The resting motor threshold (RMT) was then determined as the minimum intensity required to elicit MEPs ≥50μV (peak-to-peak amplitude between 20 and 50 ms after stimulus onset) in the contralateral FDI in at least 5 out of 10 consecutive trials.

### Continuous theta burst stimulation (cTBS)

The stimulation consisted of bursts of three pulses delivered at 50 Hz and repeated at 5 Hz for a total of 600 pulses (duration: 40 s) [2]. Contrasting with several previous studies (e.g. [2, 4, 14]), the intensity of the TMS pulses used to deliver cTBS was set to 80% of the RMT, rather than 80% of the active motor threshold (AMT). This was justified by our experience that the within-subject variability of the RMT is lower than that of the AMT, and by previous results showing that TBS delivered using 80% of the RMT generates similar effects as TBS delivered using 80% of the AMT [23].

### Motor-evoked potentials (MEPs)

The intensity of the single pulses used to elicit MEPs and TEPs was set to 110% of the RMT [16]. Biphasic pulses were chosen over monophasic pulses because previous studies [24] have shown that, following biphasic stimulation, recovery to baseline of the EEG signals can occur as soon as 10–12 ms after the onset of the TMS pulse. In contrast, following monophasic stimulation the EEG signal was found to remain offset by several μV for more than 50 ms. The current direction was the same as that used to deliver cTBS. The electromyogram (EMG) was recorded from the left and right FDI muscles using pairs of disposable Ag-AgCl surface electrodes. The signals were amplified and filtered (bandwidth 3 Hz– 20 kHz) using a Digitimer NeuroLog System (Digitimer, UK) and digitized at 5 kHz using a CED Micro 1401–3 (Cambridge Electronic Design, UK). For each trial, MEP amplitude was defined as the peak-to-peak amplitude of the maximal EMG response between 20 and 50 ms after stimulus onset. MEP latency was defined as the time point at which the EMG signal exceeded by more the 5 times the standard deviation the EMG signal measured during the pre-stimulus interval (-200 to 0 ms before the onset of the TMS pulse).

### TMS-evoked brain potentials (TEPs)

The single pulses used to elicit MEPs were also used to identify TMS-evoked brain potentials in the ongoing EEG recording. After re-referencing the signals to the average of the left and right mastoids (M1M2) [16], a linear interpolation of the signals sampled between -5 ms and +15 ms relative to the onset of the TMS pulse was used to remove pulse and step response artifacts. Then, the signals were filtered with a 1–100 Hz zero phase 4^th^ order Butterworth band-pass filter and a 50 Hz notch filter. After segmentation (from -500 ms to +1000 ms) and baseline correction (reference interval: -500 ms to -10 ms), epochs with artifacts including eye-movements were rejected based on visual inspection. Separate average waveforms were then obtained for each time point (T0, T1, T2) and condition (TMS delivered over the ipsilateral or contralateral hemisphere). Within the obtained waveforms, five distinct peaks were identified at electrode Cz: P30, N40, P60, N100 and P190 [16]. This electrode was chosen because (1) previous studies have shown that the main component of TEPs (N100-P190) is maximal at electrode Cz [25,26,16], (2) the other peaks of TEPs (P30, N40, P60) can also be measured reliably at electrode Cz [16], and (3) the signals recorded from electrodes located directly under the coil are often contaminated by large TMS-related artifacts.

### Somatosensory-evoked brain potentials (SEPs)

The electrical stimuli used to elicit SEPs consisted of 0.5 ms constant current square-wave pulses generated using a DS7 stimulator (Digitimer Ltd, UK) and delivered over the left and right median nerve at the level of the wrist using disposable adhesive electrodes. The intensity of stimulation was set to 2x the motor threshold, which was defined as the intensity required to elicit a small but consistent and visible twitch of the thumb. Using a common reference, a linear interpolation of the signals sampled between -2 ms and +10 ms was used to the electrical stimulation artifact. Then, the signals were filtered with a 0.3 Hz zero phase 4^th^ order Butterworth high-pass filter and a 50 Hz notch filter. After segmentation (from -50 ms to +150 ms) and baseline-correction (reference interval: -50 ms to -5 ms). Epochs with amplitude values exceeding ±100 μV were automatically rejected. Separate average waveforms were then obtained for each time point and condition. Within the individual average waveforms, seven distinct peaks were identified at the central electrode contralateral to the stimulated hand (left hand: C4; right hand: C3): P15, N20, P27, N30, P45 and N60. To account for possible differences in baseline drifts across conditions, peak amplitudes were estimated by measuring peak-to-peak amplitudes relative to the early-latency P15 wave. Considering that the P15 reflects subthalamic stimulus-evoked activity, it can be expected to be largely unaffected by cTBS [27].

### Statistical analyses: group-level effects

The group-level effects of cTBS on the responses obtained at the different time points and conditions were assessed using a 3-way repeated-measures ANOVA with the factors ‘time’ (T0, T1 vs. T2), TMS ‘current direction’ (AP-PA vs. PA-AP) and ‘hemisphere’ (MEPs or TEPs elicited by stimulation of the ipsilateral vs. contralateral hemisphere relative to the hemisphere onto which cTBS was applied; SEPs recorded from the central electrode ipsilateral vs. contralateral relative to the hemisphere onto which cTBS was applied). The Mauchley's test was used to evaluate the sphericity assumption. When required, results were corrected using the Greenhouse–Geisser procedure.

The presence of a three-way interaction between the factors ‘time’, ‘current direction’ and ‘hemisphere’ was considered as evidence that cTBS delivered in the AP-PA vs. PA-AP direction exerted a significantly different effect on the responsiveness of the ipsilateral vs. contralateral sensorimotor cortex. The presence of a two-way interaction between ‘time’ and ‘hemisphere’ was considered as evidence that cTBS did not similarly affect the ipsilateral and contralateral sensorimotor cortex, but that this effect was independent of ‘current direction’. In contrast, a two-way interaction between ‘time’ and ‘current direction’ was considered as evidence that cTBS similarly affected the ipsilateral and contralateral sensorimotor cortex, but that this effect was dependent on ‘current direction’. Finally, the presence of a main effect of ‘time’ was interpreted as reflecting unspecific effects of cTBS and/or unrelated effects due, for example, to habituation or changes in vigilance. When a significant effect of the factor ‘time’ was found, separate post-hoc 3-way ANOVAs were performed for each of the two time points (T1 vs. T0 and T2 vs. T0), in order to assess the time course of the observed effect.

Furthermore, to better characterize cTBS-induced changes in the magnitude of the later, longer-lasting components of the SEP and TEP waveforms, we also performed a point-by-point repeated-measures ANOVA using each sample of the recorded waveforms. The analysis was performed using the same three factors: ‘time’ (T0, T1 vs. T2), TMS ‘current direction’ (AP-PA vs. PA-AP) and ‘hemisphere’.

### Statistical analyses: MEP latency to predict the individual effect of cTBS

To examine whether the latency of the MEPs recorded *before* applying cTBS predicted the individual effect of cTBS on sensorimotor cortex excitability, we computed, for each hemisphere and condition, the correlation between MEP latency at T0 and the MEP, TEP and SEP responses obtained at T1 and T2, using the Pearson correlation coefficient.

When significant correlations were observed, we also assessed whether the correlation coefficients were significantly different across hemispheres and conditions [28]. Intraclass correlation coefficients (ICCs) were used to assess the subject-dependence of MEP latencies.

Finally, to better assess the relationship between MEP latencies and the cTBS-induced changes in the magnitude of the later, longer-lasting components of the SEP and TEP waveforms, we also computed a point-by-point correlation between MEP latency at T0 and each sample of the difference waveforms obtained by subtracting the TEP and SEP waveforms obtained at T0 from the TEP and SEP waveforms obtained at T1 and T2.

## Results

### Group-level effect of cTBS

#### MEPs

**Fig 2:**
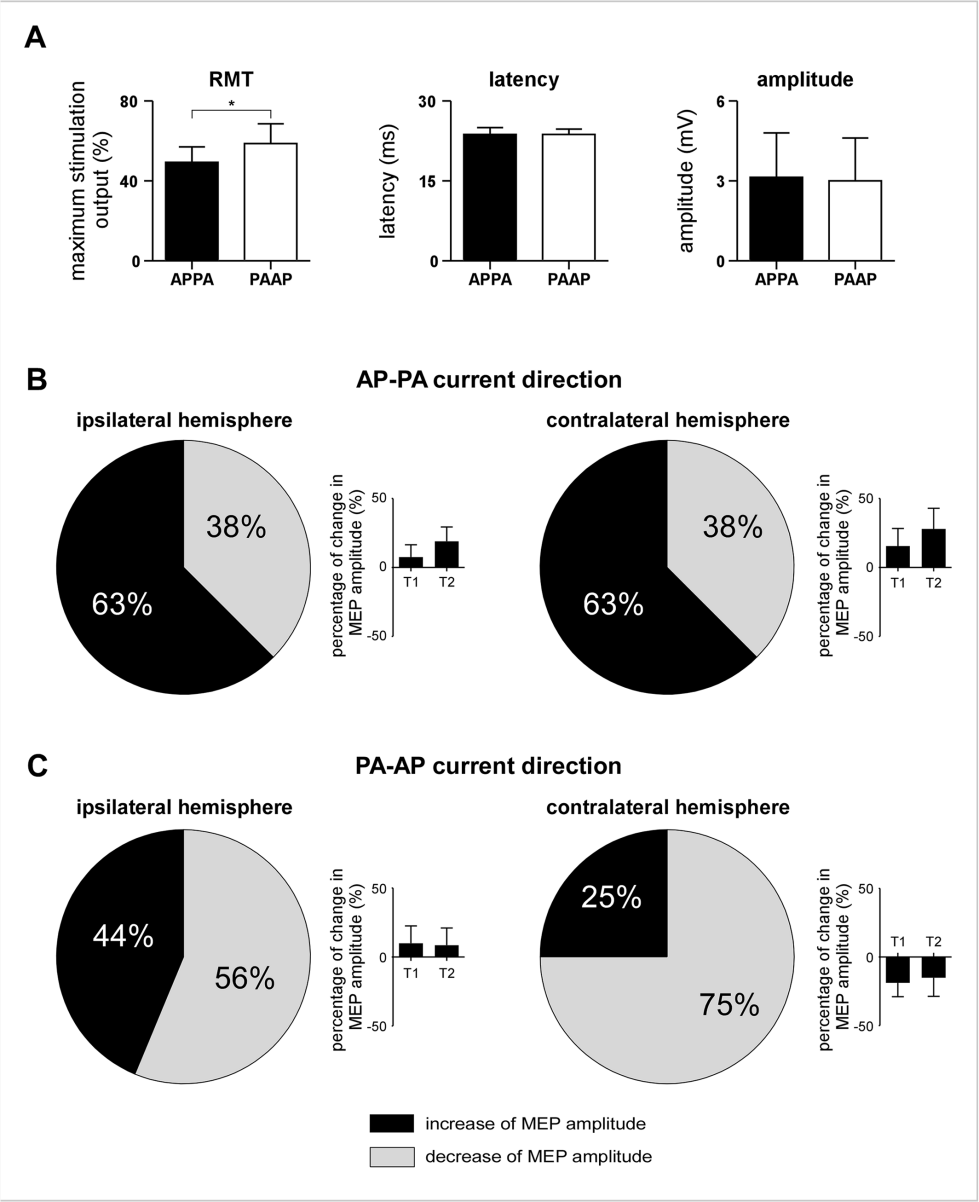
Group-level effects of cTBS on the magnitude of MEPs. (A) cTBS was delivered over M1, using biphasic pulses with an AP-PA or PA-AP current direction. The resting motor threshold (RMT) was significantly lower for TMS pulses delivered in the AP-PA as compared to the PA-AP direction (p<10^−5^; paired-sample t- test). The latency and amplitude of the MEPs elicited at baseline by AP-PA vs. PA-AP pulses delivered using an intensity of 120% the RMT were not significantly different. (B) Following cTBS delivered in the AP-PA current direction, the magnitude of MEPs elicited by stimulation of the ipsilateral and contralateral M1 tended to increase at both time points (T1 and T2). However, this effect was highly variable across individuals. (C) Similarly, following cTBS delivered in the PA-AP direction, the magnitude of MEPs elicited by stimulation of the ipsilateral and contralateral hemisphere tended to decrease in most participants. However, this effect was also highly variable across individuals. On average, there was no significant group-level effect of cTBS on MEP magnitude.

The average RMT, and the average amplitude and latencies of the MEPs obtained at time point T0 are shown in Fig 2A. As expected, the RMT was significantly greater when TMS was delivered in the AP-PA direction as compared to the PA-AP direction (paired t-test: p<10^−5^). The amplitude and latencies of the MEPs elicited by AP-PA vs. PA-AP stimulation at 120% of the RMT were not significantly different (amplitude: p = .677; latency: p = .340).

The group-level average magnitude of the MEPs obtained at the different time points and conditions are shown in Fig 2B and S1 Table. The three-way repeated-measures ANOVA revealed no significant interaction between the factors ‘time’, ‘current direction’ and ‘hemisphere’ (time*hemisphere: F = 3.00, p = .065; time*current direction*hemisphere: F = 3.62, p = .060; Table 1). Furthermore, the 3-way repeated-measures ANOVA did not reveal any significant differences between the MEP latencies.

**Table 1:** Effect of cTBS on the magnitude of MEPs, TEPs and SEPs recorded from the ipsilateral and contralateral hemisphere (3-way ANOVA with the factors time [T0, T1, T2], current direction [AP-PA, PA-AP] and hemisphere [ipsilateral or contralateral relative to the hemisphere onto which cTBS was applied]).

		time			time*current direction			time*hemisphere			time* current direction* hemisphere		
		F	p	partial η^2^	F	p	partial η^2^	F	p	partial η^2^	F	p	partial η^2^
MEP		0.55	.581	.036	2.43	.105	.140	3.00	.065	.167	3.62	.060	.195
TEP	P30	1.45	.251	.088	0.89	.387	.056	0.49	.615	.032	0.39	.619	.025
	N40	1.17	.325	.072	0.40	.586	.026	0.77	.421	.049	0.36	.704	.023
	P60	0.93	.406	.058	0.36	.628	.023	0.51	.529	.033	0.42	.661	.027
	N100	**7.16**	**.010** **	**.323**	0.30	.746	.019	1.62	.216	.097	0.55	.585	.035
	P190	0.74	.486	.047	0.84	.404	.053	0.38	.688	.025	0.42	.660	.027
SEP	P15-N20	0.42	.661	.027	0.82	.449	.052	0.65	.479	.042	**7.43**	**.002** *	**.331**

* p<0.05

** p<0.01

#### TEPs

**Fig 3:**
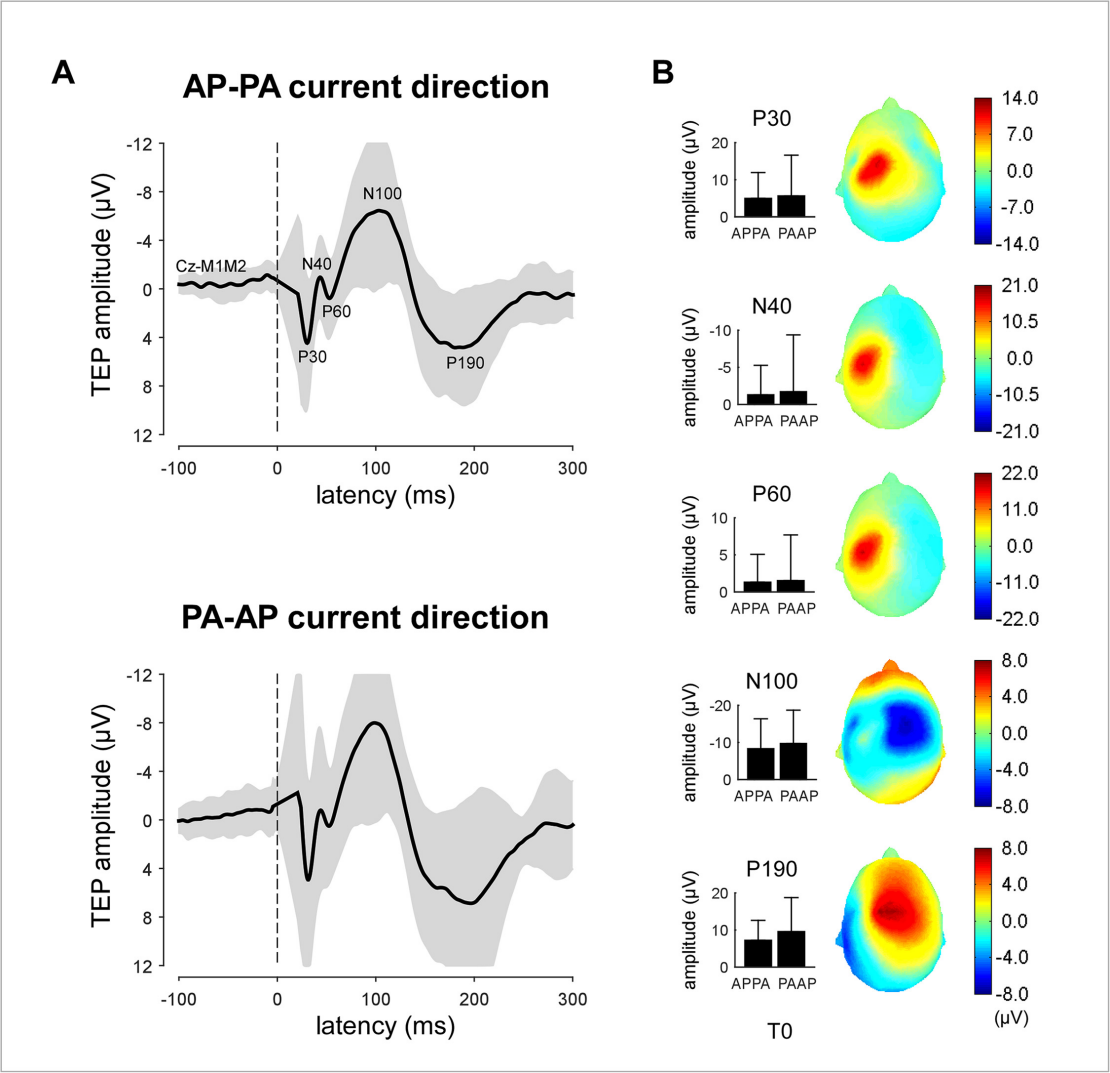
TEPs elicited by TMS delivered over M1 at T0. (A) Group-level average waveforms of the TEPs recorded before applying cTBS, and elicited by TMS pulses delivered in the AP-PA and PA-AP current direction. The grey areas represent the standard-deviation across individuals. Five peaks were consistently identified: P30, N40, P60, N100 and P190. (B) Group-level average magnitude (±standard deviation) and scalp topography of the P30, N40, P60, N100 and P190 elicited by TMS pulses delivered in the AP-PA and PA-AP directions.

The group-level average waveforms of the TEPs recorded before applying cTBS are shown in Fig 3A. At electrode Cz, five peaks were consistently identified in the single-subject waveforms: P30, N40, P60, N100 and P190 (Fig 3A). As shown in Fig 3B and S1 Table, there was no significant difference between the magnitude of the peaks elicited by TMS delivered in the AP-PA vs. PA-AP direction (paired-sample t-test).

**Fig 4:**
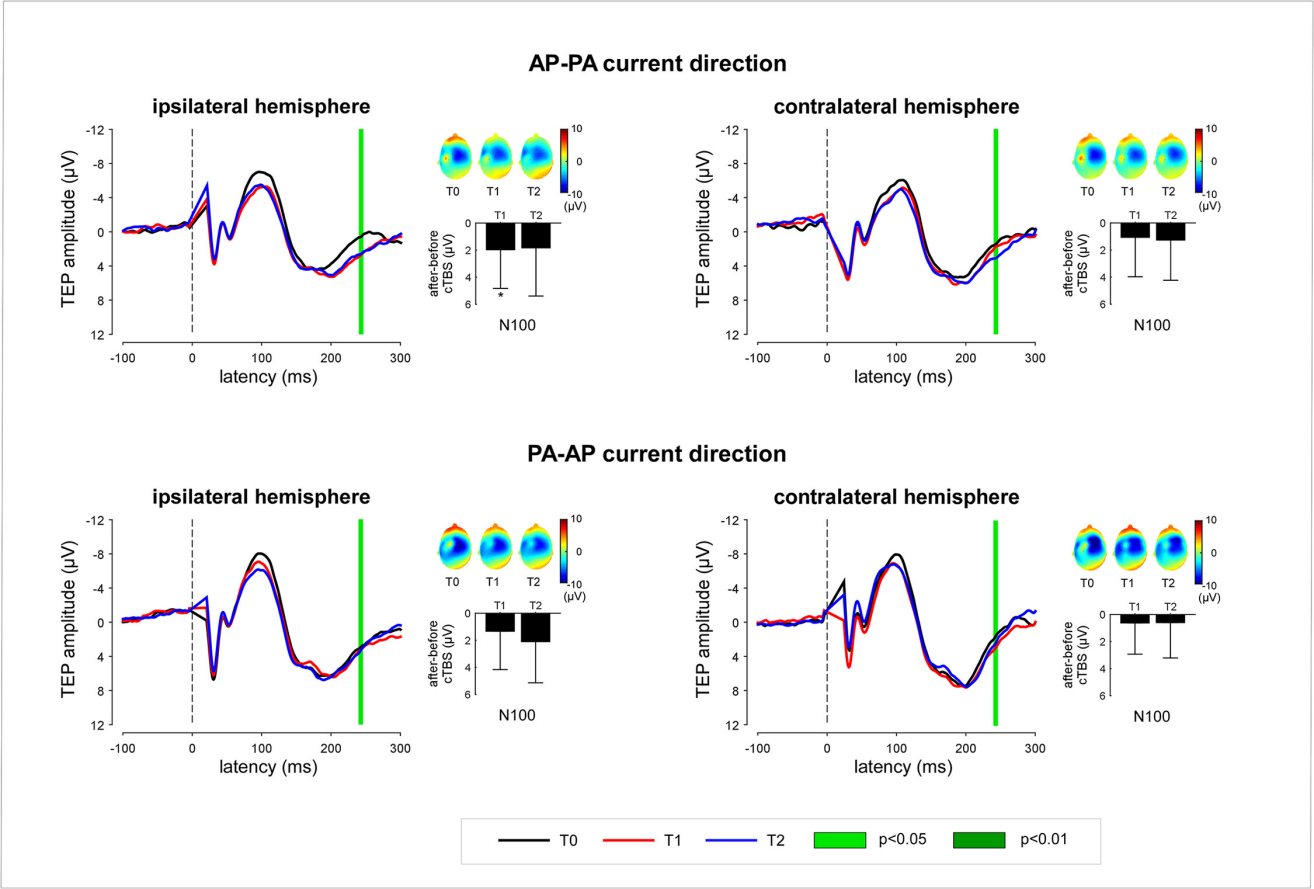
Group-level average TEP waveforms recorded before (T0) and after (T1, T2) cTBS. On average, the magnitude of the N100 wave was decreased at both T1 and T2, regardless of the TMS current direction (AP-PA vs. PA-AP) and regardless of whether cTBS was delivered over the ipsilateral or contralateral hemisphere. A point-by-point repeated measures ANOVA with the factors ‘time’ (T0, T1, T2), ‘hemisphere’ (cTBS delibered to the ipsilateral or contralateral hemisphere) and ‘current direction’ was used to assess the effect of cTBS on the entire TEP waveform. The scalp maps show the topographical distribution of the N100 at the different times points and in the different conditions. The bar graphs show the change in N100 magnitude after cTBS (group-level average ± standard deviation). Significant changes are marked by an asterisk (p < .05; t-test against zero).

The group-level average waveforms of the TEPs obtained at the different time points and in the different conditions are shown in Fig 4. The 3-way repeated-measures ANOVA only revealed a significant main effect of ‘time’ on the magnitude of the N100 wave (F = 7.16, p = .003), without any significant interaction with the factors ‘current direction’ and ‘hemisphere’ (Table 1). Post-hoc comparisons of N100 magnitude showed that it was significantly reduced both at T1 vs. T0 (F = 8.04, p = .0125) and T2 vs. T0 (F = 7.81, p = .0136). The point-by-point ANOVA of the entire TEP waveforms confirmed this main effect of time on the later component of the TEP waveforms, and also showed no significant long lasting interaction between the factors ‘time’, ‘current direction’ and ‘hemisphere’.

#### SEPs

**Fig 5:**
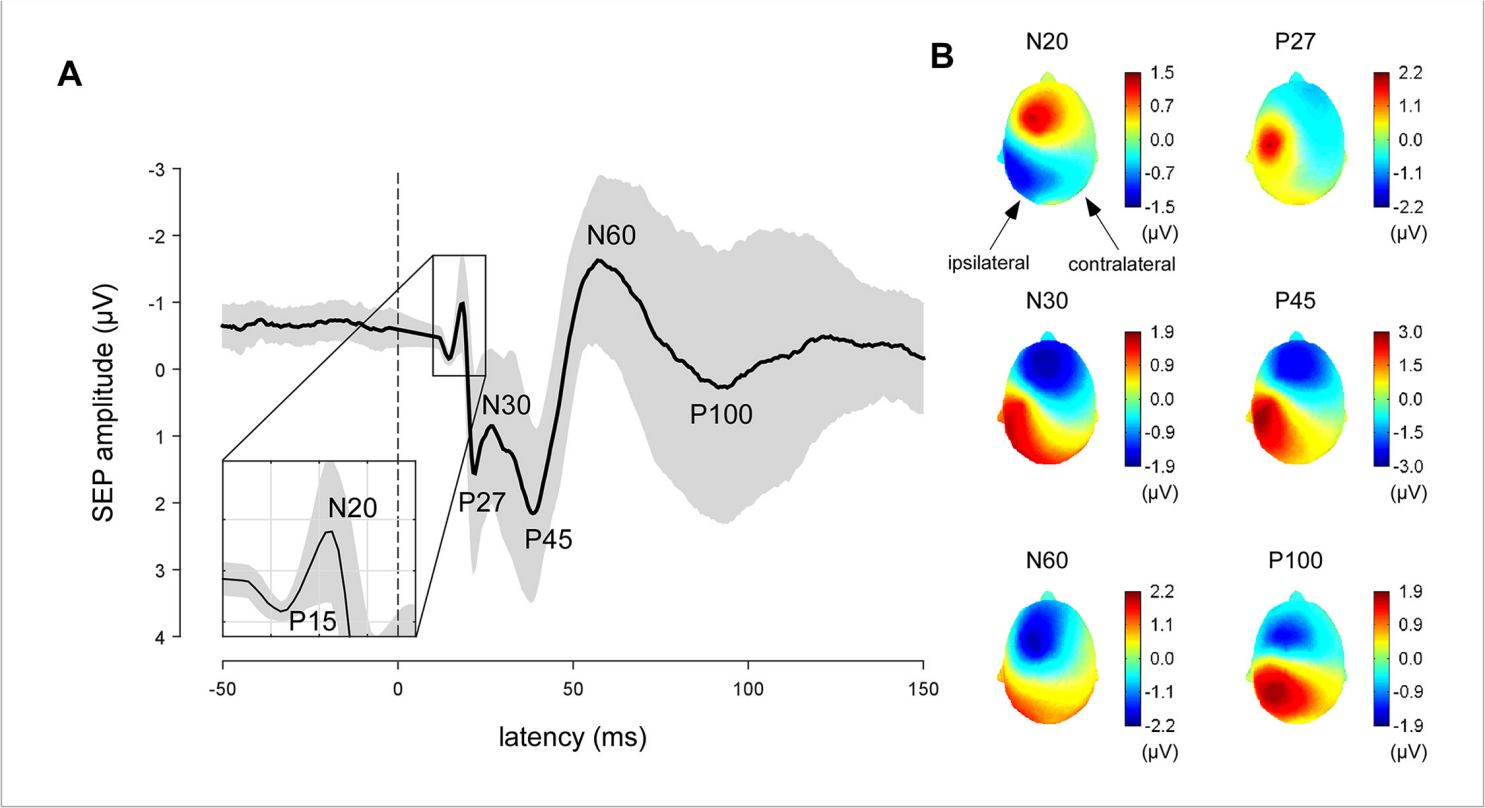
SEPs elicited by stimulation of the median nerve at T0. (a) Group-level average waveforms of the SEPs recorded before applying cTBS. The shaded areas represent the standard-deviation across individuals. Seven peaks were consistently identified at the central electrode contralateral to the stimulated hand (C3/C4): P15, N20, P27, N30, P45, N60 and P100. (b) Group-level average scalp topographies of the N20, P27, N30, P45, N60 and P100 peaks, averaged across conditions.

The group-level average waveforms of the SEPs recorded before applying cTBS are shown in Fig 5A. At the central electrode (C3/C4) contralateral to the stimulated hand, seven peaks were consistently identified in the single-subject waveforms: P15, N20, P27, N30, P45, N60 and P100. The corresponding topographical distributions are shown in Fig 5B.

**Fig 6:**
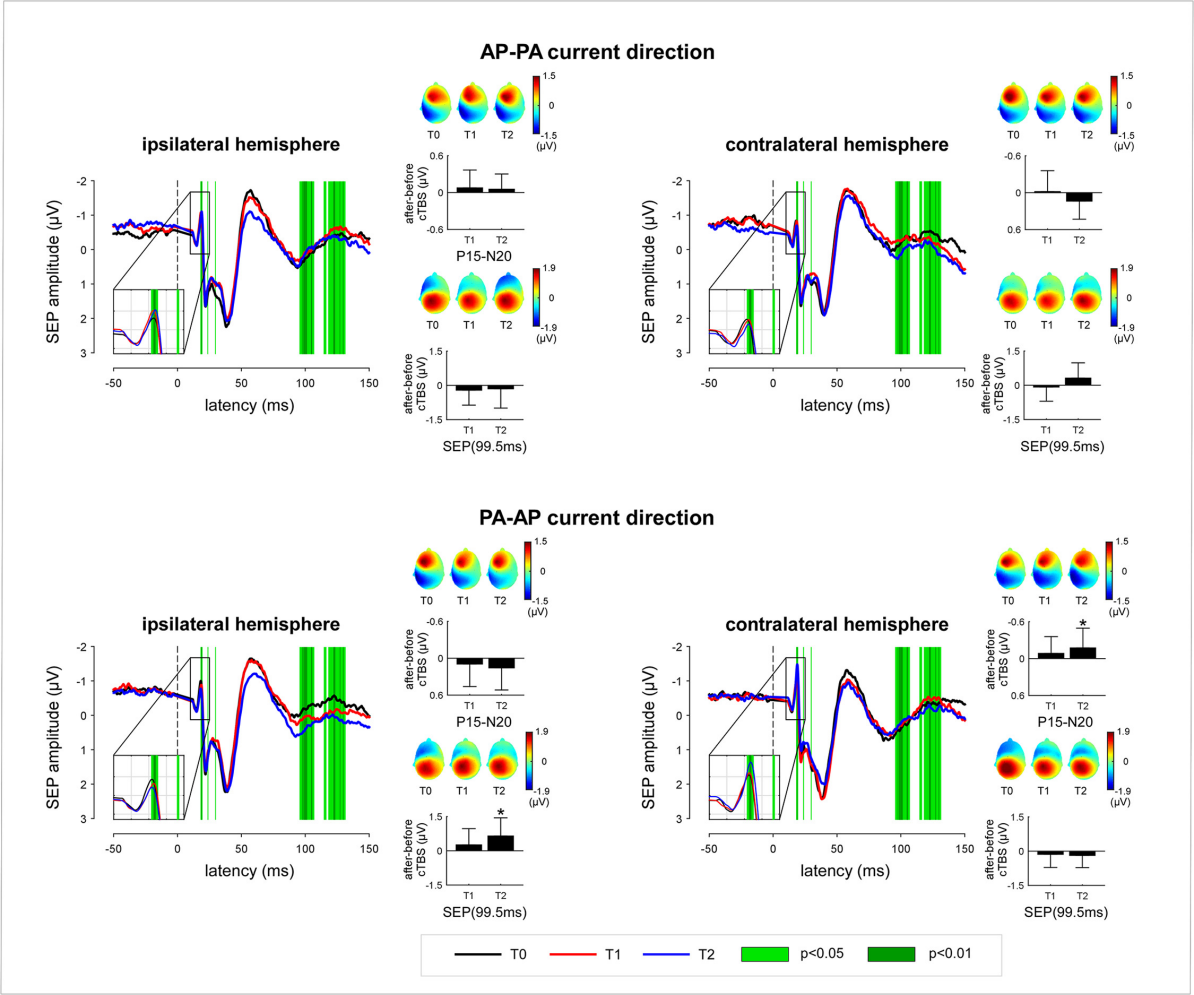
Group-level average SEP waveforms recorded before (T0) and after (T1, T2) cTBS. SEPs recorded from the ipsilateral/contralateral hemisphere were elicited by stimulation of the contralateral/ipsilateral hand, relative to the hemisphere onto which cTBS was applied. A point-by-point repeated measures ANOVA with the factors ‘time’ (T0, T1, T2), ‘hemisphere’ (cTBS delibered to the ipsilateral or contralateral hemisphere) and ‘current direction’ was used to assess the effect of cTBS on the entire SEP waveform. The time intervals showing a significant 3-way interaction between the three factors are shown in green. This included the N20 wave, as well as a longer-lasting period encompassing the late P100 wave. The bar graphs represent the change in magnitude of the N20 wave as well as the late P100 (99.5 ms) (group-level average ± standard deviation). Significant changes are marked by an asterisk (p < .05; t-test against zero). The scalp maps show the topographical distribution of the N20 and later P100 at the different times points and in the different conditions.

The group-level average SEP waveforms obtained at the different time points and in the different conditions are shown in Fig 6. The 3-way repeated-measures ANOVA revealed a significant interaction between the factors ‘time’, ‘current direction’ and ‘hemisphere’ for the P15-N20 (F = 7.43, p = .002; Table 1). Post-hoc comparisons showed that, after cTBS, the three-way interaction effect on the magnitude of the N20 was not significant at T1 vs. T0 (F = 3.23, p = .092), whereas it was significant at T2 vs. T0 (F = 18.01, p = .001). As shown in Fig 6, when cTBS was delivered in the AP-PA direction, the magnitude of the N20 tended to increase over the ipsilateral hemisphere, whereas it tended to decrease over the contralateral hemisphere. Conversely, when cTBS was delivered in the PA-AP direction, the N20 tended to decrease over the ipsilateral hemisphere and to increase over the contralateral hemisphere. The point-by-point ANOVA of the entire SEP waveforms confirmed a significant interaction between the factors ‘time’, ‘current direction’ and ‘hemisphere’ at the latency of the N20 wave, but also during a long time interval encompassing the late P100 wave. As shown in Fig 6, when cTBS was delivered in the AP-PA direction, the magnitude of the SEP at that latency tended to decrease over the ipsilateral hemisphere, whereas it tended to increase over the contralateral hemisphere. Conversely, when cTBS was delivered in the PA-AP direction, the magnitude of the SEP at that latency tended to increase over the ipsilateral hemisphere and to decrease over the contralateral hemisphere.

### MEP latency to predict the inhibitory or excitatory effects of cTBS

#### MEPs

**Fig 7:**
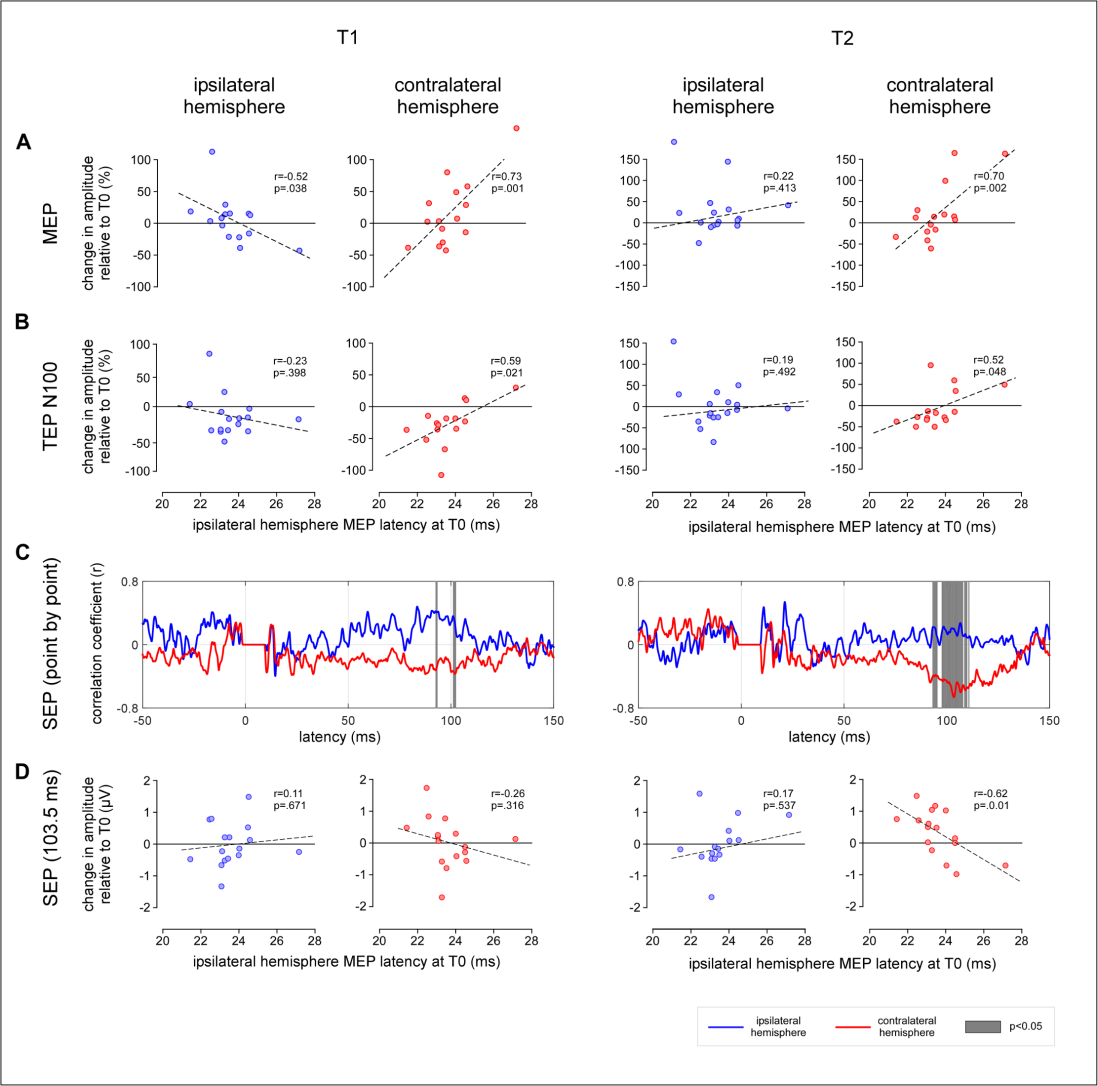
Relationship between MEP latency at T0 and the after-effect of cTBS on the magnitude of MEPs, TEPs and SEPs ipsilateral and contralateral hemisphere relative to the hemisphere onto which cTBS was applied. (TMS delivered using an AP-PA current direction). (A) At the ipsilateral hemisphere, there was a significant negative correlation between MEP latency at T0 and change in MEP amplitude. Also note, at the contralateral hemisphere, the significant positive correlation between MEP latency and MEP amplitude, as well as (B) TEP N100 amplitude. (C) Finally, note the inverse correlation between MEP latency and the magnitude of the SEP waveform recorded from the ipsilateral and contralateral hemisphere, extending between approximately 50–130 ms, both at T1 and at T2. The grey areas mark the time intervals during which the correlation coefficients obtained at the ipsilateral and contralateral hemisphere were significantly different (p<0.05). (D) At 103.5 ms, the negative correlation between MEP latency at T0 and change in SEP amplitude arrives maximum.

The relationship between single-subject MEP latencies obtained before applying cTBS (T0) and the effects of cTBS on MEP amplitude at time point T1 is shown in Fig 7.

When TMS was delivered in the AP-PA direction, there was a significant *negative* correlation between MEP latency and the change in amplitude of the MEPs elicited by stimulation of the ipsilateral hemisphere (r = -0.52, p = .038): short MEP latencies at T0 were associated with an enhancement of MEP amplitude whereas long MEP latencies at T0 were associated with a reduction of MEP amplitude. Contrasting with this negative correlation, there was a significant *positive* relationship between MEP latency and the change in the amplitude of the MEPs elicited by stimulation of the contralateral hemisphere (r = 0.73, p = .001): short MEP latencies at T0 were associated with a reduction of MEP amplitude, whereas long MEP latencies at T0 were associated with an enhancement of MEP amplitude. The correlation coefficients of the relationships between MEP latency and the amplitude of the MEPs elicited by stimulation of the ipsilateral and contralateral hemispheres were significantly different (p = .0001).

A trend in the same direction was observed when TMS was delivered in the PA-AP direction. However, the correlation between MEP latencies at T0 and the changes in MEP amplitudes at T1 were not significant (Table 2).

**Table 2:** Correlation between MEP latency at T0 and the after-effect of cTBS delivered using an AP-PA or PA-AP current direction on the magnitude of MEPs, TEPs (N100), and SEPs (at 103.5ms) recorded from the ipsilateral or contralateral hemisphere relative to the hemisphere onto which cTBS was applied.

		AP-PA				PA-AP			
		ipsilateral		contralateral		ipsilateral		contralateral	
		r	p	r	p	r	p	r	p
MEP	T1	**-0.52**	**0.038** *	**0.73**	**0.001** **	-0.12	0.658	0.11	0.699
	T2	0.22	0.413	**0.70**	**0.002** **	0.17	0.526	-0.06	0.812
TEP N100	T1	-0.23	0.398	**0.59**	**0.021** *	-0.27	0.330	-0.33	0.245
	T2	0.19	0.492	**0.52**	**0.048** *	-0.14	0.631	0.23	0.448
SEP P100	T1	0.11	0.671	-0.26	0.316	-0.13	0.623	0.04	0.896
	T2	0.17	0.537	**-0.62**	**0.010** *	-0.01	0.965	-0.30	0.257

* p<0.05

** p<0.01

Similarly, at time point T2, the correlation between MEP latencies at T0 and the effect of cTBS on MEP amplitude was not as strong as at time point T1 (Fig 7). Nevertheless, when TMS was delivered in the AP-PA direction, there was still a significant positive correlation between MEP latency and the amplitude of the MEPs obtained by stimulating the contralateral hemisphere (r = 0.70, p = 0.002).

#### TEPs

At both time points following cTBS (T1 and T2), there was a significant relationship between MEP latencies obtained before applying cTBS and the post-effect of cTBS on the absolute magnitude of the N100 peak (Fig 7).

When TMS was delivered in the AP-PA direction, there was no significant correlation between MEP latency and the change in amplitude of the N100 elicited by stimulation of the ipsilateral hemisphere (T1: r = -0.23, p = .398; T2: r = 0.19, p = .492). However, there was a significant *positive* correlation between MEP latency and the change in amplitude of the N100 elicited by stimulation of the contralateral hemisphere, both at T1 (r = 0.59, p = .021) and at T2 (r = 0.52, p = .048). Short MEP latencies at T0 were associated with a reduction of the contralateral N100, whereas long MEP latencies at T0 were associated with an enhancement of the contralateral N100. The correlation coefficients of the relationships between MEP latency and the amplitude of the MEPs elicited by stimulation of the ipsilateral and contralateral hemispheres were significantly different at T1 (p = .022), but not at T2 (p = .394).

No significant relationship between MEP latency and change in amplitude of the other peaks of the TEP waveform was observed.

#### SEPs

When TMS was delivered in the AP-PA direction, there was no significant correlation between the latency of the MEPs recorded at baseline, and the change in amplitude of each peak of the SEP waveform (N20, P27, N30, P45, N60). However, the point-by-point correlation analysis of the SEP waveforms showed, both at T1 and at T2, a positive relationship between MEP latency and the magnitude of the late positive SEP wave recorded over the ipsilateral hemisphere following stimulation of the contralateral hand and, conversely, a negative relationship between MEP latency and the magnitude of that same late positive SEP wave recorded over the contralateral hemisphere following stimulation of the ipsilateral hand (Fig 7). The correlation coefficients of the relationships between MEP latency and the amplitude of the SEPs recorded from the ipsilateral and contralateral hemispheres were significantly different at both time points.

When cTBS was delivered in the PA-AP direction, there was no significant correlation between the latency of the MEPs recorded at baseline, and the change in magnitude of the SEP waveforms.

### Time, subject, hemisphere dependence of MEP latency

**Fig 8:**
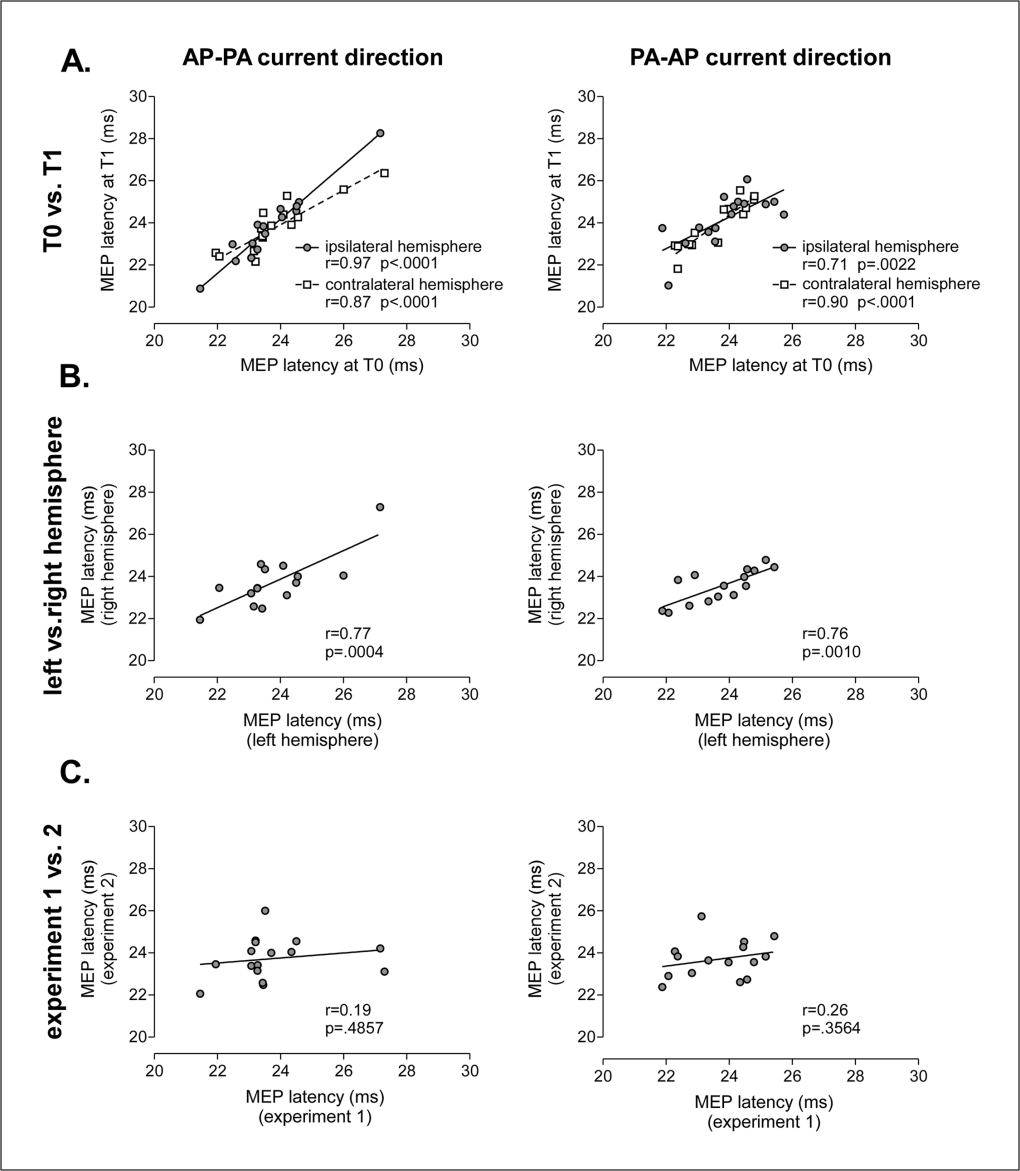
Relationship between the latency of MEPs obtained at different time points and in different experiments. (A) Relationship between MEP latencies recorded at T0 and MEP latencies recorded at T1, following stimulation of the ipsilateral and contralateral hemisphere. (B) Relationship between MEP latencies elicited by stimulation of the left and right M1. C. Relationship between MEP latencies recorded at T0 in each of the two experiments, separated by 14–91 days. Left graphs, TMS delivered using an AP-PA current direction. Right graphs: TMS delivered using a PA-AP current direction.

Each participant took part in four different experiments, in which cTBS was delivered over the left or right hemisphere, using TMS pulses delivered in an AP-PA or PA-AP current direction. In each experiment, MEPs were recorded following stimulation of the left and right M1 before applying cTBS. This allowed us to assess whether the variability of MEP latency is subject-dependent, hemisphere-dependent and/or time-dependent–i.e. whether MEP latency is determined by intrinsic subject or hemisphere characteristics (e.g. M1 cortex geometry) or whether it is determined by time-dependent changes in brain function (e.g. context-dependent changes in M1 cortex excitability or connectivity) (Fig 8).

There was a significant intraclass correlation between MEP latencies and subjects (ICC = 0.75, p = .002), indicating that MEP latencies are strongly dependent on subject-dependent factors. This could have been in part related to a relationship between MEP latency and peripheral conduction distance. However, there was no significant correlation between MEP latency and standing height (r = 0.56, p = .146).

Within a given experiment, there was a highly-significant within-subject correlation between MEP latencies recorded at T1 and T0, both when TMS was delivered in the AP-PA direction (IPSI: r = 0.97, p < .0001; CON: r = 0.87, p < .0001) and in the PA-AP direction (IPSI: r = 0.70, p < .0022; CON: r = 0.90, p < .0001). There was also a strong within-subject correlation between the MEP latencies obtained following stimulation of the left and right M1 (AP-PA: r = 0.77, p = .0004; PA-AP: r = 0.76, p = .001).

## Discussion

### Modulatory effects of cTBS on the ipsilateral motor cortex

Our results confirm that the after-effect of cTBS applied over M1 on motor cortex excitability is highly variable across individuals [2,29,30] (Fig 2), and that this inter-individual variability can be predicted by the latency of the MEPs recorded before applying cTBS [4] (Fig 7).

Using monophasic TMS pulses, Hamada et al. [4] showed a significant correlation between the after-effects of cTBS and the latency of MEPs elicited by pulses delivered in the AP current direction (r = -0.686; p<10^−7^), especially when latencies were expressed relative to the latency of MEPs elicited by pulses delivered in the lateral-medial (LM) direction (r = -0.712; p<10^−8^). Computing this latency difference was justified by the notion that high-intensity pulses delivered in the LM direction evokes an early latency response related to the direct activation of pyramidal cell axons. Hence, the AP-LM latency difference could constitute a more reliable measure of the differential recruitment of cortical interneurons, as compared to the absolute AP latency. The finding that the effects of cTBS on MEP amplitude may be predicted by the latency of the MEPs recorded before applying cTBS has thus been interpreted as resulting from the fact that both MEP latency and the effects of cTBS on M1 excitability are dependent on the population of interneurons preferentially recruited by the TMS pulse.

In the present study, single pulses of TMS were delivered as single biphasic pulses either in the AP-PA direction or the PA-AP direction. When TMS was delivered in the AP-PA direction, there was a significant negative correlation between MEP latency at T0 and the after-effect of cTBS on the amplitude of the MEPs elicited by stimulation of the ipsilateral M1 (T1: r = 0.73, p = .001). Such as in Hamada et al. [4], when MEP latencies were short, cTBS tended to increase the magnitude of the MEPs elicited at T1. In contrast, when MEP latencies were long, cTBS tended to reduce the magnitude of the MEPs elicited at T1. A similar negative relationship between MEP latency and the after-effects of cTBS was observed when TMS was delivered in the PA-AP direction. However, this relationship was not significant, confirming the notion that cTBS exerts a more consistent effect on M1 when it is delivered in the AP-PA direction [31].

The threshold to elicit responses using biphasic TMS is, on average, lower than the threshold to elicit responses using monophasic TMS [32]. Direction specific effects of AP-PA biphasic pulses are usually considered to resemble those of monophasic PA pulses, whereas the direction-specific effects of PA-AP pulses are considered to resemble those of AP pulses [32]. However, comparison of the descending volleys elicited by biphasic and monophasic pulses, Di Lazzaro et al. [33] showed that biphasic pulses produce a more complex pattern of activation than monophasic pulses, indicating that both phases of biphasic TMS can elicit responses, and that the precise combination of elements activated by each phase depends on their relative threshold and the relative amplitude of each phase. Therefore, our finding that the correlation between the latency of MEPs and the after-effect of cTBS was stronger when the TMS pulses were delivered in the AP-PA direction as compared to the PA-AP direction could be mainly related to the fact that the threshold to elicit motor responses was lower in the AP-PA direction as compared to the PA-AP direction (Fig 2; [32]).

Several factors could explain why TMS may preferentially activate different interneuronal networks across individuals and, hence, explain the variability of MEP latency as well as the variability of the after-effects of cTBS. This variability could be explained by inter-individual variations in the size, shape and orientation of M1. Alternatively, the variability could be explained by fluctuations in the state of the stimulated brain region, leading different neuronal populations to be differently sensitive to cTBS. The relative contribution of these two factors can be assessed by examining the within-subject correlation of MEP latencies across sessions of a given experiment performed on the same day and across different experiments separated by several days. Indeed, inter-individual variability related to structural differences can be expected to remain constant across time and, hence, to remain constant across different experiments. In contrast, inter-individual variability related to changes in brain function can be expected to vary as a function of time, depending on the state of the subject.

In the present study, each participant took part in several experiments, separated by several days. Within a given experiment, there was a very strong within-subject correlation between the latencies of the MEPs recorded in each of the three experimental sessions. Furthermore, within each session, there was a strong correlation between the latencies of the MEPs elicited by stimulation of the left and right hemisphere. Finally, there was no significant correlation between the latencies of the MEPs recorded in the two separate experiments, which were separated by at least 14 days (14–91 days). Supporting this result, Hamada et al. [4] found a consistent within-subject relationship between MEP latencies recorded in two experiments separated by a shorter number of days. However, Vernet et al. [29] found that, with longer intervals (107 ±177 days), the after-effects of cTBS on MEP magnitude varied greatly within the same individual. The finding that the intra-class correlation of MEP latency is time-dependent (being strong when the recordings are repeated at short time intervals and weaker when the recordings are repeated at long intervals) indicates that the inter-individual variability of MEP latency as well as the inter-individual variability of the after-effects of cTBS are not only determined by fixed neuroanatomical factors (e.g. structural difference in M1 configuration). Instead, it suggests that the variability of MEP latencies and variability of the effects of cTBS are at least partly related to time-varying changes in M1 function.

### Modulatory effects of cTBS on the contralateral motor cortex

Contrasting with the *negative* correlation between MEP latency and the post-effects of cTBS on the excitability of the ipsilateral motor cortex, we found a significant *positive* correlation between MEP latency and the post-effects of cTBS on the excitability of the *contralateral* motor cortex. Indeed, when the latency of the MEPs elicited by stimulation of the ipsilateral hemisphere at T0 was short, cTBS tended to decrease the magnitude of the MEPs elicited by stimulation of the contralateral hemisphere, both at T1 and at T2 (Fig 7). In contrast, when MEP latencies were long, cTBS tended to increase the magnitude of the MEPs elicited at T1 and T2.

The finding that MEP latencies at T0 predict opposite effects of cTBS on the excitability of the ipsilateral and contralateral motor cortex agrees with the well-established notion of inter-hemispheric inhibition between the left and right M1 [34]. Indeed, depending on whether cTBS increases or decreases the excitability of the ipsilateral M1, inter-hemispheric inhibition would be expected to lead to a relative decrease or increase of the excitability of the contralateral M1. Similar findings have been reported in other studies [12–14]. For example, Di Lazzaro et al. [35] compared the effects of cTBS and iTBS delivered over M1 in healthy individuals and patients with acute stroke. He did not observe any significant difference between the group-level effects of cTBS and iTBS, but found that the individual effects of cTBS on the contralateral M1 were reversed as compared to those on the ipsilateral M1.

### TMS-evoked brain potentials

The functional significance of the ERP waveforms that can be elicited by TMS pulses delivered over M1 remains poorly understood [19]. Most importantly, whether they truly reflect cortical activity elicited by the direct stimulation of M1 remains an open question. First, at least part of the elicited waveforms could be contaminated by TMS-induced artifacts such as electrode polarization artifacts due to the capacitive properties of the electrode contact, electrode movement artifacts and muscle activity artifacts due to stimulation of cranial or facial muscles. Furthermore, because the TMS pulse generates a loud sound as well as a tactile sensation at the site of stimulation, the elicited waveforms could receive a contribution of auditory and/or somatosensory ERPs.

In the present study, group-level comparisons showed a main effect of time on the magnitude of the N100 wave: on average, the magnitude of the N100 wave recorded at T1 and T2 was reduced as compared to T0. This time-dependent habituation of the N100 wave, which was also reported by Vernet et al. [36], indicates that the N100 does not reflect a stimulation artifact generated by the TMS pulse. However, it does not exclude the possibility that the N100 reflects auditory- and/or somatosensory-evoked brain activity.

Paus et al. [37] showed a significant correlation between the magnitude of MEPs and the magnitude of the N100 wave of TEPs. This observation suggests that the N100 is truly related to the stimulation of M1, but could also be explained by the fact that TMS pulses generating a large MEP are also more likely to generate a stronger auditory and/or tactile stimulus.

In the present study, we show a significant relationship between MEP latencies measured at T0 and the effects of cTBS on the magnitude of the N100 wave measured at both T1 and T2 (Fig 7). When TMS was delivered in the AP-PA direction, short MEP latencies at T0 predicted a reduction of N100 amplitude, whereas long MEP latencies at T0 predicted an enhancement of N100 amplitude. No such relationship was observed when TMS was delivered in the PA-AP direction. These observations constitute first-time evidence that the N100 wave is directly related to the stimulation of M1 and, hence, that TEPs can be used to probe changes in functional connectivity within the human brain.

### Modulatory effects of cTBS on the primary somatosensory cortex

The group-level analysis of the effects of cTBS on the magnitude of SEPs showed that cTBS delivered using the AP-PA current direction led to an increase of the magnitude of the N20 wave recorded over the *ipsilateral* hemisphere, whereas cTBS delivered using the PA-AP current direction led to an increase of the magnitude of the N20 wave recorded over the *contralateral* hemisphere. This finding contradicts to the results of previous studies showing that cTBS delivered over M1 does not affect S1[38,39].

In addition to this group-level effect, we also found an inverse relationship between the latency of the MEPs elicited at T0 and the change in magnitude of the later portion of the SEP waveforms obtained from the ipsilateral and contralateral hemisphere (Fig 7). Most interestingly, this relationship was reversed as compared to the relationship between MEP latency and the effects of cTBS on MEP amplitude and TEP N100 amplitude. Over the ipsilateral hemisphere, short-latency MEPs at T0 tended to be associated with a reduction of the SEPs, and an enhancement of the MEPs and TEPs. Conversely, over the contralateral hemisphere, short-latency MEPs tended to be associated with an enhancement of the SEPs, and a decrease of the MEPs and TEPs.

Our results support the results of previous studies suggesting that cTBS delivered over M1 exerts opposing effects on M1 and S1 (i.e. that cTBS delivered over M1 tends to increase the excitability of M1 and, conversely, to decrease the excitability of S1) [40]. These opposing effects could be explained by sensorimotor inhibitory interactions and/or the existence of inter-hemispheric inhibitory interactions between the left and right S1 [41].

## Limitations

In the present study, the effects of cTBS delivered over M1 using an AP-PA current direction were tested by measuring MEPs and TEPs elicited by TMS pulses also delivered in the AP-PA direction; whereas the effects of cTBS delivered using the PA-AP direction were tested using TMS pulses delivered in the PA-AP direction. Hence, the results of the present study cannot disentangle effects of the current direction used to deliver cTBS from effects of current direction used to elicit the MEPs.

## Conclusion

Our results confirm that the highly variable after-effects of cTBS delivered over the primary sensorimotor cortex can be predicted by the latency of the MEPs recorded before applying cTBS. When MEPs exhibit short latencies, cTBS tends to increase the excitability of the ipsilateral M1, and to decrease the excitability of the contralateral M1, as evidenced by its effect on the amplitude of MEPs as well as the amplitude of the N100 wave of TEPs. Conversely, when MEPs exhibit later latencies, cTBS tends to decrease the excitability of the ipsilateral M1 and to increase the excitability of the contralateral M1. Most interestingly, the reverse effect was found when assessing the relationship between MEP latencies and the excitability of S1, as demonstrated by the concurrent recording of SEPs. Future studies should examine whether MEP latencies can predict the clinical response of repetitive TMS used to treat neurological and psychiatric disorders.

## Supporting Information

S1 Dataset The data for MEP, TEP and SEP. MEP, SEP and TEP data were converted to .xls format from .mat format.
(ZIP)

S1 Table The mean values for all the measurement at T0 in different conditions of current direction and hemisphere (current direction: AP-PA, PA-AP, hemisphere: ipsilateral or contralateral relative to the hemisphere onto which cTBS was applied). No significant difference has been checked out for any measurement in the two way repeated measure ANOVA (current direction * hemisphere).
(DOCX)
