# Supplementary material for: MEP Latencies Predict the Neuromodulatory Effect of cTBS Delivered to the Ipsilateral and Contralateral Sensorimotor Cortex
Source: PLoS One. 2015 Aug 11;10(8):e0133893. doi: 10.1371/journal.pone.0133893 (PMC4532482; doi:10.1371/journal.pone.0133893)
Supplement: S1 Table — No significant difference has been checked out for any measurement in the two way repeated measure ANOVA (current direction * hemisphere). (DOCX) [file pone.0133893.s002.docx]

| amplitude  (at T0) | | MEP | TEP | | | | | SEP |
| --- | --- | --- | --- | --- | --- | --- | --- | --- |
|  |  |  | **P30** | **N40** | **P60** | **N100** | **P190** | **P15-N20** |
| APPA | **IPSI** | 3.26 | 4.14 | -1.41 | 1.09 | -8.93 | 6.57 | -1.30 |
|  | **CON** | 3.02 | 5.83 | -1.19 | 1.55 | -7.71 | 7.91 | -1.23 |
| PAAP | **IPSI** | 2.59 | 7.10 | -0.87 | 1.35 | -9.80 | 9.38 | -1.41 |
|  | **CON** | 3.42 | 4.20 | -2.56 | 1.72 | -9.61 | 9.80 | -1.56 |
